# Comparison of outcomes of peritoneal dialysis between patients after failed kidney transplant and transplant-naïve patients: a meta-analysis of observational studies

**DOI:** 10.1080/0886022X.2021.1914659

**Published:** 2021-04-26

**Authors:** Xiaohua Meng, Weifei Wu, Shuang Xu, Zhiqun Cheng

**Affiliations:** Department of Nephrology, Huzhou Central Hospital, Affiliated Central Hospital of HuZhou University, Huzhou, Zhejiang Province, P.R. China

**Keywords:** Renal replacement therapy, renal allograft, mortality, dialysis, kidney transplant, peritonitis

## Abstract

**Purpose:**

The influence of prior failed kidney transplants on outcomes of peritoneal dialysis (PD) is unclear. Thus, we conducted a systematic review and meta-analysis to compare the outcomes of patients initiating PD after a failed kidney transplant with those initiating PD without a prior history of kidney transplantation.

**Methods:**

We searched PubMed, Embase, CENTRAL, and Google Scholar databases from inception until 25 November 2020. Our meta-analysis considered the absolute number of events of mortality, technical failures, and patients with peritonitis, and we also pooled multi-variable adjusted hazard ratios (HR).

**Results:**

We included 12 retrospective studies. For absolute number of events, our analysis indicated no statistically significant difference in technique failure [RR, 1.14; 95% CI, 0.80–1.61; I^2^=52%; *p* = 0.48], number of patients with peritonitis [RR, 1.13; 95% CI, 0.97–1.32; I^2^=5%; *p* = 0.11] and mortality [RR, 1.00; 95% CI, 0.67–1.50; I^2^=63%; *p* = 0.99] between the study groups. The pooled analysis of adjusted HRs indicated no statistically significant difference in the risk of technique failure [HR, 1.25; 95% CI, 0.88–1.78; I^2^=79%; *p* = 0.22], peritonitis [HR, 1.04; 95% CI, 0.72–1.50; I^2^=76%; *p* = 0.85] and mortality [HR, 1.24; 95% CI, 0.77–2.00; I^2^=66%; *p* = 0.38] between the study groups.

**Conclusion:**

Patients with kidney transplant failure initiating PD do not have an increased risk of mortality, technique failure, or peritonitis as compared to transplant-naïve patients initiating PD. Further studies are needed to evaluate the impact of prior and ongoing immunosuppression on PD outcomes.

## Introduction

Chronic kidney disease with an estimated global prevalence at 13.4% continues to be a major public health problem. Studies indicate that approximately 4.902 to 7.083 million patients have end-stage renal disease (ESRD) and require renal replacement therapy (RRT) [1]. Allograft survivals after kidney transplantations have improved, but a single kidney transplant seldom meets the life-long RRT requirements of most patients [2,3]. According to one study, the 10-year graft failure rates after deceased-donor and live-donor transplantations are 42.3% and 26.5%, respectively [4]. Moreover, the relisting rates for repeat kidney transplants are resulting in many patients with failed kidney transplants returning to dialysis [5,6].

Perl et al. [7] in their retrospective review of 16 113 patients demonstrated that the use of peritoneal dialysis (PD), when compared to hemodialysis (HD), may improve the outcomes in patients after a failed kidney transplant. However, whether the PD outcomes in this cohort are different from those of transplant-naïve patients initiating PD remains unclear. Research indicates that mortality and morbidity in dialysis patients after allograft failure are high when compared to those of patients awaiting kidney transplants [8]. This has been attributed to the different characteristics between patients with failed transplants and transplant-naïve patients. Failed allografts can generate a persistent alloimmune reaction in affecting clinical outcomes. Moreover, the prolonged immunosuppressant therapy in patients with a kidney transplant, which sometimes is continued after graft failure, may also influence clinical outcomes [9].

Studies have compared PD outcomes between patients after kidney transplant failure and patients initiating PD without any history of kidney transplant. However, the evidence has been conflicting with studies from different geographical regions and variable sample sizes [10,11]. To the best of our knowledge, no systematic review has synthesized the available data to present high-level evidence for the clinicians managing patients with failed kidney transplants. Therefore, we systematically searched the literature and conducted a meta-analysis to compare clinical PD outcomes between patients initiating PD after failed kidney transplant and transplant naïve patients.

## Materials and methods

### Inclusion criteria

We followed the PRISMA statement guidelines (Preferred Reporting Items for Systematic Reviews and Meta-analyses) [12] to generate this review. However, the study protocol was not registered. We defined the inclusion criteria based on PICOS (Population, Intervention, comparison, outcomes, study type). Criteria for each domain was as follows:

*Population*: ESRD patients on PD
*Intervention*: Patients with failed kidney allograft (Tx group)
*Comparison*: Transplant naïve patients (*ie*, patients who had not had a kidney transplant) (nTx group)
*Outcomes*: Studies reporting data on at least one of the following three outcomes: mortality, technique survival, and peritonitis. Technique survival was defined as the transfer of the patient to HD.

#### Study type: all types of studies

We excluded the following studies: 1) Studies comparing outcomes of HD and PD after kidney transplant. 2) Studies failing to report the exact RRT used after failed kidney transplant. 3) Studies failing to report relevant outcomes. 4) Review articles, non-English language studies, and case reports. For studies reporting duplicate or overlapping data, we included the study with the larger sample size.

### Search strategy

Based on the inclusion criteria, two independent reviewers conducted an electronic search in the PubMed, Embase, CENTRAL, and Google Scholar databases from inception to 25 November 2020 to identify relevant publications. They used the following keywords in different combinations: ‘kidney transplant’, ‘prior kidney transplant’, ‘failed kidney transplant’, ‘transplant naive’, ‘failed renal allograft’, ‘prior renal allograft’, ‘failure’, ‘peritoneal dialysis’, and ‘renal replacement therapy’. Supplementary Table S1 depicts the search strategy. Two reviewers independently evaluated the titles and abstracts of search results and then the full texts of relevant publications. All full-texts were reviewed based on the inclusion and exclusion criteria and only articles satisfying all the criteria were finally selected for this review. Any disagreements were resolved by discussion. To avoid missing relevant studies, the reviewers manually reached the bibliographies of included studies for any additional references.

### Data extraction and risk of bias assessment

At the beginning of the review, we extracted detailed data from the studies onto a prepared form to gather first author, publication year, study location, sample size, demographic details of patients, comorbidities (diabetes mellitus and cardiovascular disease), baseline estimated glomerular filtration rate, baseline hemoglobin and albumin, number of patients on automated PD, patients on immunosuppressant therapy, study outcomes, and follow-up information. The outcomes of interest were the difference in technique failure, peritonitis rates and mortalities between the study groups.

We assessed the quality of the studies using the risk of a bias assessment tool for non-randomized studies (RoBANS) [13]. Two reviewers assessed the selection of participants, confounding variables, intervention measures, blinding of outcome assessment, incomplete outcome data, and selective outcome reporting in each study.

### Statistical analysis

We used the ‘Review Manager’ (RevMan, version 5.3; Nordic Cochrane Center [Cochrane Collaboration], Copenhagen, Denmark; 2014) for the meta-analysis. We extracted two types of values for the outcomes of interest. First, we extracted the absolute numbers of fatal events, technique failures, and patients with peritonitis and pooled the data to calculate risk ratios (RR) with 95% confidence intervals (CI). We performed a sub-group analysis based on the baseline matching carried out by the study authors for the study groups. Next, we extracted multivariable-adjusted hazard ratios (HR) for the three outcomes and we used the generic inverse function of the meta-analysis software to pool them. We chose a random-effects model for the meta-analysis because all the studies included were retrospective and heterogenous. We calculated the *I^2^* statistic to assess inter-study heterogeneity (values between 25 and 50% represented low heterogeneity, values between 50 and 75% represented moderate heterogeneity, and values higher than 75% represented substantial heterogeneity). We used funnel plots to assess publication bias.

## Results

**Figure 1.**
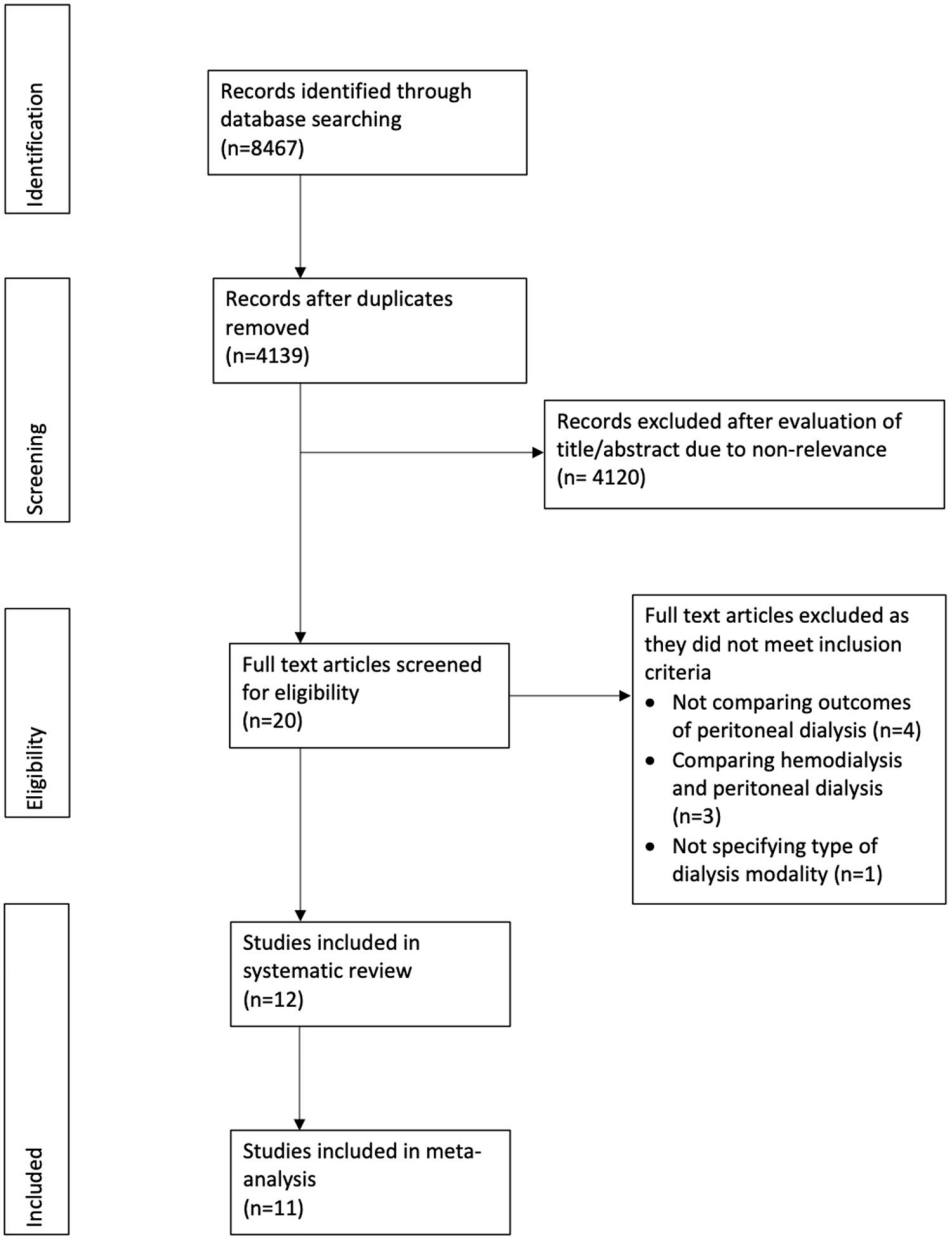
Study flow chart.

Figure 1 presents the study flow-chart. We identified 20 studies for full text analysis of which 12 fulfilled the inclusion criteria [10,11,14–23]. Table 1 lists the details of all the studies included (all retrospective in nature and reporting data from different world regions). The number of patients in the Tx groups varied from 28 to 494, while that in the nTx group varied from 43 to 13 638. Five studies matched the study cohorts based on baseline variables. We found wide variation in the follow-ups of the included studies. Only five studies reported data on immunosuppressive therapy in the Tx cohort (Supplementary Table S2). Steroids were commonly used in the early PD periods in the Tx cohort, but we found wide variations in the percentages of patients on immunosuppressant drugs.

**Table 1.** Details of included studies.

Study	Study type	Study location	Sample size	Mean age (y)	Male gender (%)	DM (%)	CVD (%)	eGFR (ml/min/1.73 m^2^)	Hb (g/dL)	Albumin (g/dL)	APD (%)	Follow-up
De Costa 2020 [11]	RT*	Brazil	Tx : 47 nTx : 47	46.1 ± 12.1 47 ± 12.7	38 38	68 68	66 70	6 (4–10)^$^ 8.2 (6–11.1)^$^	10.5 ± 2 10.8 ± 1.8	3.4 ± 0.45 3.8 ± 0.45	85 85	14.8 (9.4–30.7)m^$^ 23.5 (2–21.2)m^$^
Benomar 2018 [10]	RT*	France	Tx : 328 nTx : 656	50.7 (19.5–82.6)^ 50.8 (18.2–91.4)^	50.6 52	13.7 24.7	NR	NR	NR	NR	64.9 45.7	17 (14–20)m^#^ 21. (19–23)^#^
Chaudhri 2016 [14]	RT*	UK	Tx : 50 nTx : 93	40.4 ± 1.8 42.8 ± 1.3	60 59	NR	NR	NR	10.5 ± 0.2 11 ± 0.2	3.87 ± 0.08 4.01 ± 0.05	NR	26m
Han 2015 [15]	RT	Korea	Tx : 41 nTx : 712	40.1 ± 11.2 NR	68.3 NR	14.6 NR	NR	NR	10.8 ± 1.71 NR	3.6 ± 0.59	24.4	Up to 8y
Yang 2013 [16]	RT	Korea	Tx : 47 nTx : 668	40.8 ± 10.7 51 ± 14.2	59.6 54.8	NR	NR	NR	NR	NR	NR	Up to 10y
Chen 2012 [17]	RT	USA	Tx : 445 nTx : 2384	NR NR	53 52.9	NR	NR	NR	NR	NR	NR	Up to 3y
Najafi 2012 [18]	RT	Iran	Tx : 43 nTx : 1067	37.4 ± 14.6 45.9 ± 21.1	62 42	25.5 30.9	NR	NR	NR	3.5 ± 0.5 3.6 ± 0.7	NR	3–119m
Mujais 2006 [19]	RT*	USA	Tx : 494 nTx : 491	39.7 ± 14.6 39.7 ± 14.6	48.6 48.5	25.3 24.9	NR	NR	NR	NR	65 65	Up to 5y
Badve 2006 [20]	RT	Australia & New Zealand	Tx : 309 nTx : 13638	37.6 ± 16.6 57 ± 16.5	48.9 52.2	15.2 35.5	8.7 39.4	5.3 (4.1–8.4)^$^ 6.3 (4.8–8.3)^$^	NR	NR	NR	12.4 (5.3–25.4)m^$^ 15 (6.3–28.9)m^$^
Duman 2004 [21]	RT	Turkey	Tx : 34 nTx : 82	39.4 ± 2 41.6 ± 1.5	48 52	0 0	NR	NR	NR	4 ± 0.3 4.1 ± 0.2	NR	Up to 5y
Sasal 2001 [22]	RT*	Canada	Tx : 42 nTx : 43	42.2± NR 38.9± NR	64 65	20.9 23.8	NR	NR	NR	NR	NR	Up to 100m
Davies 2001 [23]	RT	UK	Tx : 28 nTx : 469	41.2± NR 54.7± NR	NR	18 18	14 25	NR	NR	NR	NR	Up to 10y

^Median (range); ^$^Median (interquartile range);^#^Mean (range).

*matching of study groups done.

RT, retrospective; DM, diabetes mellitus; CVD, cardiovascular disease; eGFR, estimated glomerular filtration rate; Hb, hemoglobin; APD, automated peritoneal dialysis; Tx, failed transplant group; nTx, non-transplant group; m, months; y, years.

Table 2 presents a descriptive analysis of study outcomes and results from all the studies included. Two studies reported higher technique failures in the Tx group than in the nTx group. A separate group of two studies reported higher mortality in the Tx group than in the nTx group. Chen et al. [21] found a borderline higher incidence of peritonitis in the Tx group than in the nTx group, while Chaudhri et al. [23] found a lower risk of peritonitis in the Tx group. Five studies reported the time-to-first peritonitis episode with four of them finding no differences between the two groups, while Badve et al. [18] found significantly longer times in the Tx group than in the nTx group. We did not conduct a meta-analysis with these data about time-to-first peritonitis episode because they had not been reported in a standard format. Table 3 presents detailed results of the quality analysis of the studies included.

**Table 2.** Outcomes and results from the included studies.

Study	Outcome	Result
De Costa 2020 [11]	Death	Significantly higher risk in the Tx group (HR: 4.4 95% CI 1.49, 13.2 p = 0.007)
	Technique failure	No significant difference between the two groups (HR: 1.14 95% CI 0.59, 2.21 p = 0.69)
	Peritonitis rate	No significant difference between the two groups (HR: 1.41 95% CI 0.78, 2.56 p = 0.25)
	Time to first peritonitis episode	No significant difference between the two groups [Tx: 9.9 (3.0–6.5) vs nTx: 7.5 (5.0–16.8) months p = 0.73]
	Cumulative risk of peritonitis	No significant difference between the two groups (HR: 1.59 95% CI 0.90, 2.82 p = 0.11)
	Type of microorganisms in the peritoneal dialysate	No significant difference between the two groups (p = 0.68)
Benomar 2018 [10]	Death	No significant difference between the two groups (Tx: 10.1% vs nTx: 12.5% p = 0.30)
	Technique failure	Significantly higher in the Tx group (Tx: 44.2% vs nTx: 30.2% p < 0.0001)
	Peritonitis rate	No significant difference between the two groups (Tx: 43.6% vs nTx: 40.1% p = 0.30)
	Time to first peritonitis episode	No significant difference between the two groups (Tx: 7.2 (0–105) vs nTx: 9.1 (0–71) months p = 0.06)
Chaudhri 2016 [14]	Death	No significant difference between the two groups (HR: 1.34 95% CI 0.72, 2.48)
	Technique failure	Significantly higher in the Tx group (HR: 1.81 95% CI 1.08, 3.0)
	Risk of peritonitis	Significantly lower in the Tx group (HR: 0.46 95% CI 0.22, 0.93)
	Peritoneal membrane function	No significant difference between the two groups
Han 2015 [15]	Peritonitis	No significant difference between the two groups (Data not reported)
	Exit site infection	Significantly higher in the Tx group (HR: 2.7 95% CI 1.51, 4.85)
Yang 2013 [16]	Death	No significant difference between the two groups (HR: 0.75 95% CI 0.45, 1.25 p = 0.26)
	Technique failure	No significant difference between the two groups (HR: 0.88 95% CI 0.53, 1.45 p = 0.61)
Chen 2012 [17]	Risk of peritonitis	Borderline higher in the Tx group (HR: 1.19 95% CI 0.99, 1.42 p = 0.06)
Najafi 2012 [18]	Death	No significant difference between the two groups (HR: 0.29 95% CI not reported p = 0.09)
	Technique failure	No significant difference between the two groups (Tx: 4.6% vs nTx: 17.5%)
Mujais 2006 [19]	Death	No significant difference between the two groups (Tx: 75.8 ± 3.3% vs nTx: 74.4 ± 3.9%)
	Technique failure	No significant difference between the two groups (Tx: 47.8 ± 3.5% vs nTx: 52.1 ± 3.6%)
Badve 2006 [20]	Death	No significant difference between the two groups (HR: 1.09 95% CI 0.81, 1.45 p = 0.58)
	Technique failure	No significant difference between the two groups (HR: 0.91 95% CI 0.75, 1.10 p = 0.31)
	Risk of peritonitis	No significant difference between the two groups (HR: 0.92 95% CI 0.72, 1.16 p = 0.44)
	Time to first peritonitis episode	Significantly longer in the Tx group (Tx: 20.4 vs nTx: 15.2 months p = 0.02)
Duman 2004 [21]	Death	No significant difference between the two groups (Tx: 5.8% vs nTx: 7.3% p > 0.05)
	Technique failure	No significant difference between the two groups (Tx: 60% vs nTx: 48% p > 0.05)
	Peritonitis rate	No significant difference between the two groups (Tx: 0.125 ± 0.025 vs nTx: 0.073 ± 0.01 per patient month p > 0.05)
	Time to first peritonitis episode	No significant difference between the two groups (Tx: 382 ± 97 vs nTx: 447 ± 78 days p > 0.05)
Sasal 2001 [22]	Death	Significantly higher in the Tx group (Tx: 28.6% vs nTx: 6.9% p < 0.01)
	Technique failure	Significantly higher in the Tx group (Tx: 35.7% vs nTx: 18.6% p < 0.01)
	Time to first peritonitis episode	Significantly earlier in the Tx group (Data not reported p = 0.02)
	Peritonitis rate	No significant difference between the two groups (Tx: 1 episode/10 months nTx 1episode/11.9 months)
Davies 2001 [23]	Death	No significant difference between the two groups (HR: 0.91 95% CI not reported p = 0.81)
	Technique failure	No significant difference between the two groups (Data not reported p = 0.57)

Tx, failed transplant group; nTx transplant-naïve group; HR, hazard ratios.

Data presented in parenthesis as reported by the included studies based on availability.

**Table 3.** Risk of bias in included studies.

Study	Selection of participants	Confounding variables	Measurement of exposure	Blinding of outcome assessment	Incomplete outcome data	Selective outcome reporting
De Costa 2020 [11]	Low risk	Low risk	Low risk	High risk	Unclear risk	Low risk
Benomar 2018 [10]	High risk	Low risk	Low risk	High risk	Unclear risk	Low risk
Chaudhri 2016 [14]	Low risk	Low risk	Low risk	High risk	Unclear risk	Low risk
Han 2015 [15]	Low risk	Low risk	Low risk	High risk	Unclear risk	Low risk
Yang 2013 [16]	Low risk	High risk	Low risk	High risk	Unclear risk	Low risk
Chen 2012 [17]	High risk	High risk	Low risk	High risk	Unclear risk	High risk
Najafi 2012 [18]	High risk	High risk	Low risk	High risk	Unclear risk	Low risk
Mujais 2006 [19]	High risk	Low risk	Low risk	High risk	Unclear risk	High risk
Badve 2006 [19]	High risk	High risk	Low risk	High risk	Unclear risk	Low risk
Duman 2004 [21]	Low risk	High risk	Low risk	High risk	Unclear risk	Low risk
Sasal 2001 [22]	Low risk	Low risk	Low risk	High risk	Unclear risk	Low risk
Davies 2001 [23]	Low risk	High risk	Low risk	High risk	Unclear risk	Low risk

##### Meta-analysis

**Figure 2.**
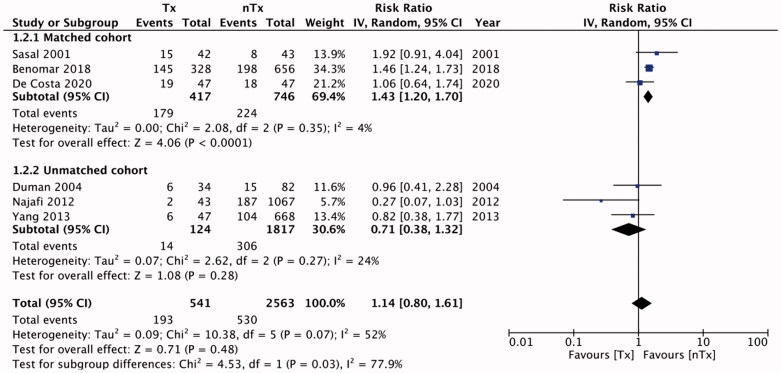
Meta-analysis of absolute technical failure events between Tx and nTx groups with sub-group analysis for matched and unmatched studies.

**Figure 3.**
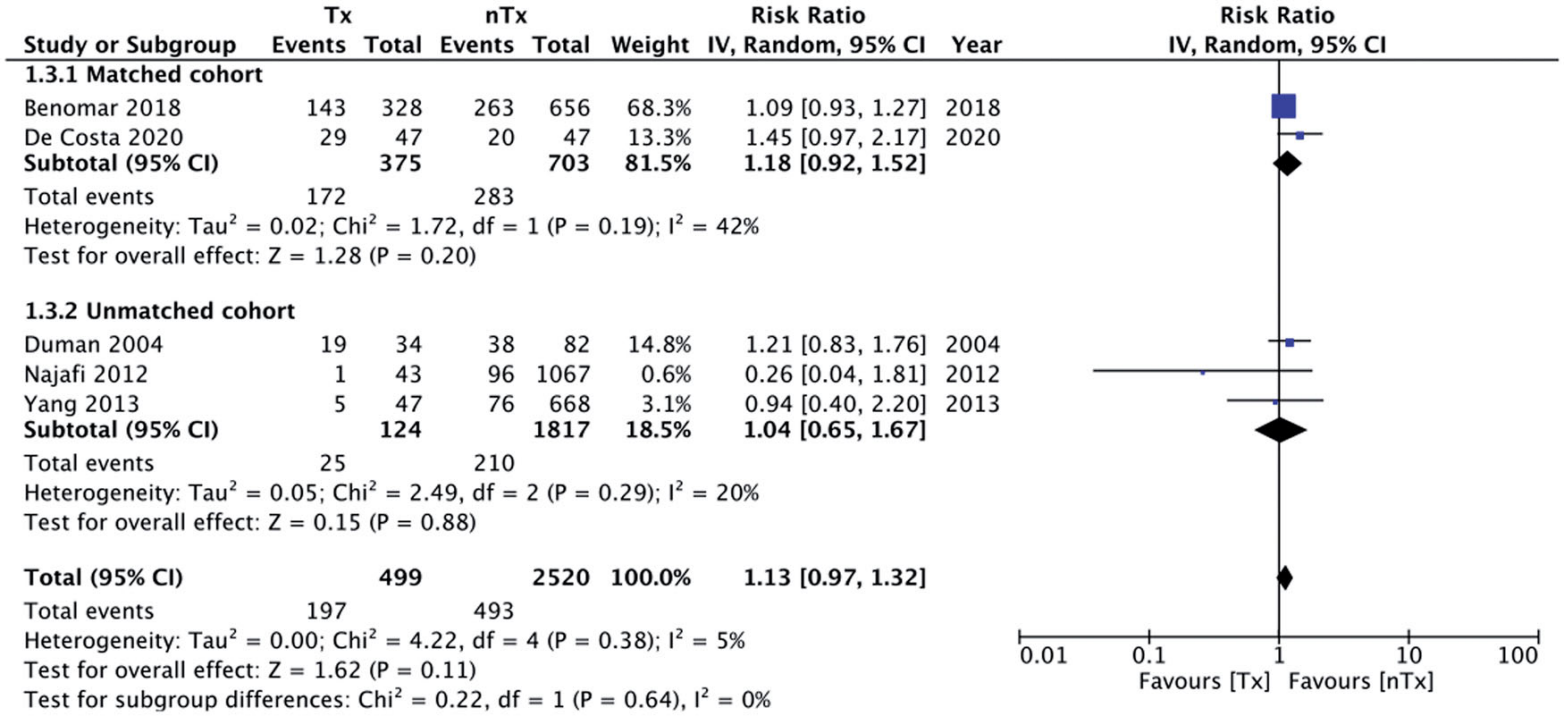
Meta-analysis of absolute number of patients with peritonitis between Tx and nTx groups with sub-group analysis for matched and unmatched studies.

**Figure 4.**
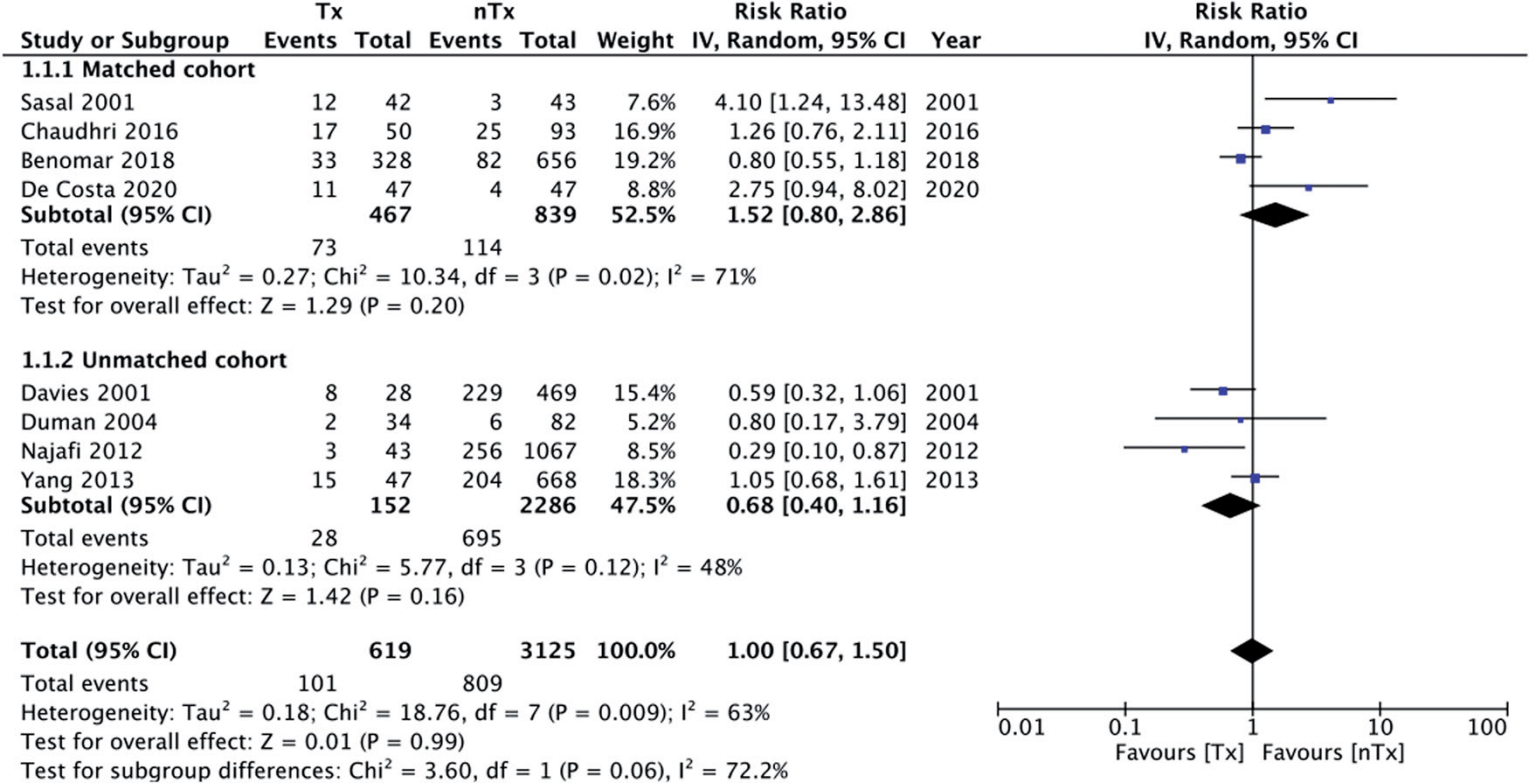
Meta-analysis of absolute mortality events between Tx and nTx groups with sub-group analysis for matched and unmatched studies.

Six studies reported data on technique failure. The rates of technique failure between the study groups were similar [RR, 1.14; 95% CI, 0.80–1.61; I^2^=52%; *p* = 0.48] (Figure 2). On subgroup analysis with pooled data for matched studies, we found technique failure to be significantly less frequent in the nTx group than in the Tx group [RR, 1.43; 95% CI, 1.20–1.70; I^2^=4%; *p* < 0.0001], but this difference was non-significant for unmatched studies [RR, 0.71; 95% CI, 0.38–1.32; I^2^=24%; *p* = 0.28]. There was no publication bias (Supplementary Figure 1). Table 4 presents our descriptive analysis of reasons for technique failure in the included studies. We found similar numbers of patients with peritonitis in the Tx and nTx groups [RR, 1.13; 95% CI, 0.97–1.32; I^2^=5%; *p* = 0.11] (Figure 3). The results were non-significant on sub-group analysis for matched [RR, 1.18; 95% CI, 0.92–1.52; I^2^=42%; *p* = 0.20] and unmatched studies [RR, 1.04; 95% CI, 0.65–1.67; I^2^=20%; *p* = 0.88]. Publication bias was not detected on funnel plot (Supplementary Figure 2). Our analysis of pooled absolute numbers of events indicated similar mortalities between the Tx and nTx groups [RR, 1.00; 95% CI, 0.67–1.50; I^2^=63%; *p* = 0.99] (Figure 4). Our results were non-significant on sub-group analyses for matched [RR, 1.52; 95% CI, 0.80–2.86; I^2^=71%; *p* = 0.20] and unmatched studies [RR, 0.68; 95% CI, 0.40–1.16; I^2^=48%; *p* = 0.16]. There was no publication bias (Supplementary Figure 3).

**Table 4:** Reasons for technique failure in the included studies.

Study	Tx group	nTx group
De Costa 2020 [11]	Peritonitis* Problems with water Catheter related Peritoneal leak Psychosocial	Peritonitis* Problems with water Catheter related Peritoneal leak Psychosocial
Benomar 2018 [10]	Ultrafiltration failure and/or Adequacy failure (47%) Peritonitis (16.8%) Malnutrition (2.8%) Catheter dysfunction, patient burn-out, inability to do PD, encapsulating peritonitis sclerosis (23.5%) Causes unrelated to PD (10.5%)	Ultrafiltration failure and/or Adequacy failure (40.4%) Peritonitis (16.7%) Malnutrition (1.5%) Catheter dysfunction, patient burn-out, inability to do PD, encapsulating peritonitis sclerosis (35.9%) Causes unrelated to PD (15.2%)
Chaudhri 2016 [14]	NR	NR
Han 2015 [15]	NR	NR
Yang 2013 [15]	Peritonitis (83.3%) Catheter related (0%) Ultrafiltration failure (0%) Others (16.7%)	Peritonitis (73.1%) Catheter related (13.5%) Ultrafiltration failure (9.6%) Others (3.8%)
Chen 2012 [17]	NR	NR
Najafi 2012 [18]	Peritonitis (50%) Catheter malfunction (0%) Exit site infection (0%) Ultrafiltration failure (0%) Mechanical problems (0%) Patient preference (50%)	Peritonitis (54%) Catheter malfunction (18.5%) Exit site infection (1.7%) Ultrafiltration failure (7.9%) Mechanical problems (0.6%) Patient preference (17.4%)
Mujais 2006 [19]	Infection (27.2%) Catheter malfunction (18.4%) Fluid management (3.9%) Ultrafiltration failure (20.4%) Psychological (5.8%) Others (22.3%)	Infection (25.2%) Catheter malfunction (18.9%) Fluid management (1.9%) Ultrafiltration failure (16.5%) Psychological (16%) Others (20.9%)
Badve 2006 [20]	NR	NR
Duman 2004 [21]	NR	NR
Sasal 2001 [22]	NR	NR
Davies 2001 [23]	NR	NR

NR, not reported; PD, peritoneal dialysis.

*Percentages not reported.

**Figure 5.**
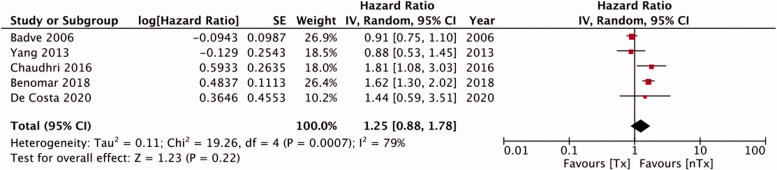
Meta-analysis of adjusted hazard ratios for technical failure.

**Figure 6.**
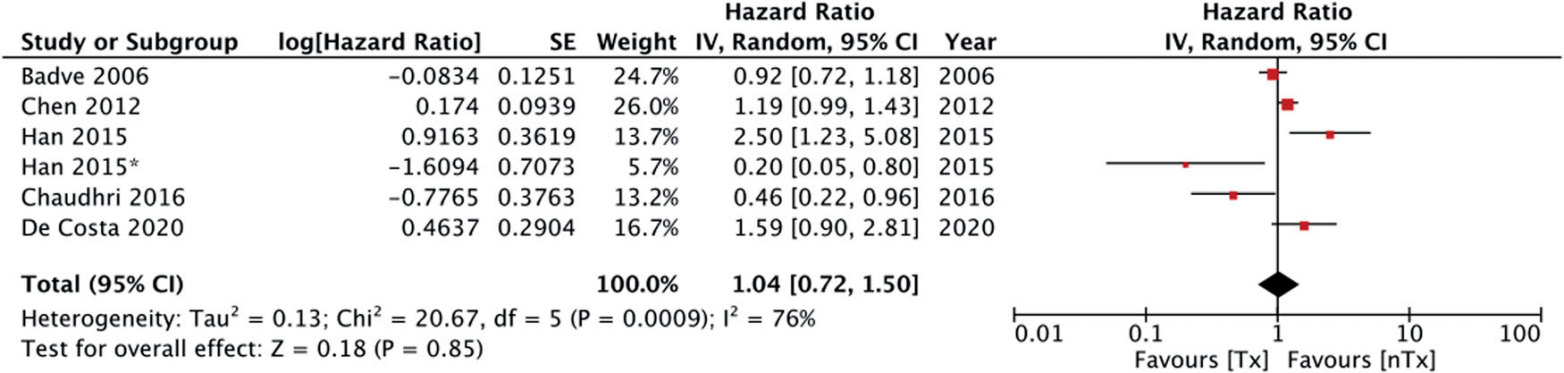
Meta-analysis of adjusted hazard ratios for risk of peritonitis.

**Figure 7.**
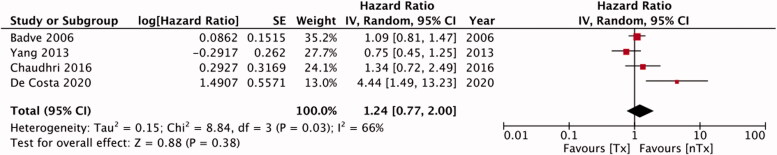
Meta-analysis of adjusted hazard ratios for mortality.

Five studies in our meta-analysis reported adjusted HRs for technique failure. We found no statistically significant differences in the risk of technique failure [HR, 1.25; 95% CI, 0.88–1.78; I^2^=79%; *p* = 0.22] between the Tx and nTx groups (Figure 5). Publication bias was not detected on funnel plot (Supplementary Figure 4). An analysis of data from five studies indicated similar risks of peritonitis in the study groups [HR, 1.04; 95% CI, 0.72–1.50; I^2^=76%; *p* = 0.85] (Figure 6). No gross asymmetry was detected on funnel plot (Supplementary Figure 5). Han et al. [14] reported outcomes of low-dose and high-dose steroid groups separately; we pooled the data from both groups for our meta-analysis. Lastly, our pooled analysis indicated similar risks of mortality between Tx and nTx groups [HR, 1.24; 95% CI, 0.77–2.00; I^2^=66%; *p* = 0.38] (Figure 7). No gross asymmetry was detected on funnel plot (Supplementary Figure 6).

## Discussion

Our results indicate that patients initiating PD after a failed kidney transplant do not have worse outcomes than transplant-naïve patients initiating PD. Our analysis indicates similar mortality, technique survival, and peritonitis rates between the two patient groups.

Kidney transplant failure is a known cause of increased morbidity and mortality in patients with ESRD. USA Renal Data System and the Canadian Organ Replacement Register data indicate that the risk of mortality in patients after allograft loss is three times higher than that of patients maintaining transplant function [24,25]. The clinical characteristics of patients on dialysis after allograft failure often differ from those of transplant-naïve patients, with studies reporting kidney function declines, and reduced hemoglobin, lower serum albumin, and increased C-reactive protein levels in these patients [26,27]. Also, the use of immunosuppression therapy further contributes to nephrotoxicity and the risk of infections in this cohort [28]. A combination of these metabolic alterations results in increased risks of cardiovascular disease, infection, and malignancy (all common causes of death) in transplant failure patients [27,29]. However, most patients returning to dialysis after a failed kidney transplant initiate HD rather than PD [10] and the studies reporting worse survivals in those patients have mostly compared HD outcomes rather than PD outcomes [8,29]. In our review, comparing PD outcomes in patients with failed transplants with those in transplant-naïve patients, we found similar mortality risks between the two groups. Our results were consistent after pooling the absolute number of events for matched and unmatched studies as well as for multivariable-adjusted HRs. The lack of difference in mortality in our analysis, despite the poor prognosis of failed kidney transplant patients [24,25] may be explained by the improved early survival offered by PD in transplant failure patients. A large study comparing outcomes of PD and HD after kidney transplant failure has reported 15% lower risk of mortality in patients initiating dialysis with PD [7]. Indeed, transplant failure patients on HD are at an increased risk of septicemia and other infectious complications especially in the first 6 months of dialysis initiation [30]. This is not only attributable to the use of immunosuppression drugs but also to the high rates of incident central venous catheter (CVC) use in transplant failure patients as compared to transplant-naïve individuals [31]. Research indicates that PD is associated with lower rates of infection-related deaths [32]. Furthermore, incident CVC use is an important cause of mortality in HD patients [33]. Thus, by initiating PD after transplant failure, patients can avoid a CVC and its associated complications. Additionally, infections with PD in transplant failure patients are usually localized with lower risk of mortality [7]. Therefore, these factors may have contributed to the better survival in transplant failure patients resulting in a non-significant result. However, while interpreting the results, it is important to note is that the follow-ups of the included studies varied widely ranging from just 3 months to a maximum of 10 years, and we could not differentiate between short-term and long-term survival. On the descriptive analysis, only one study by Sasal et al. [16] reported increased mortality in transplant failure patients than in the transplant-naïve patients. While the exact reason for this variation is difficult to explain, the small sample size of their study could have contributed to the contrasting result.

Alterations in the morphology of the peritoneal membrane leading to ultrafiltration failure are an important cause of long-term mortality in patients undergoing PD [15]. Immunosuppressant therapy is nephrotoxic in humans, but its effects on the peritoneal membrane function remain unclear [7]. Animal studies have indicated that long-term exposure to immunosuppressant therapy (calcineurin inhibition) can lead to fibrosis of the peritoneal membrane [34]. However, in one of the studies included in our review, Chaudhri et al. [23] demonstrated similar baseline dialysate-to-plasma ratios of creatinine and net ultrafiltration between the two study groups indicating a limited role for long term calcineurin inhibitors on the peritoneal membrane function in patients with kidney transplant.

In addition to ultrafiltration failure, dialysis inadequacy, and peritonitis are important causes of technique failure during PD [35,36]. These reasons were also noted in both the study groups of our review. Our descriptive analysis of the data in the studies in our review indicates that most patients were transferred to HD after technique failure. Patients with failed kidney transplants have been shown to experience a rapid decline in their residual renal function as compared to the patients who have never undergone a kidney transplant [15,37]. However, Bernando et al. [38] failed to demonstrate any deterioration in residual renal function in patients initiating PD after failed kidney transplant when compared to the residual renal function in transplant-naïve patients. Concurring with the results of Chaudhri et al. [23] and Bernando et al. [38], we found similar incidences of ultrafiltration failure or dialysis inadequacy between the two study groups. In our pooled analysis we also found similar technique failure rates between the two groups, except for the sub-group of matched studies, which involved only three studies and which had results that were largely influenced by the study of Benomar et al. [10].

Lastly, for the incidence of peritonitis, concerns have been raised that prolonged immunosuppression in the transplanted cohort may increase the risk of infections. This is particularly important as peritonitis is known to independently increase the mortality and reduce the technique survival of patients on PD [39]. However, data on immunosuppression drugs were rarely reported in the studies included in our review. Only one study in our review assessed the association between immunosuppressant therapy and the risk of peritonitis. Han et al. [14] demonstrated that the risk of peritonitis increases only with high-dose steroid therapy and non-tapering steroid protocols, and their overall analysis indicated no difference between transplant failure and transplant-naïve groups. Our pooled analysis also failed to demonstrate any statistically significant difference in the number of patients with peritonitis in either study group. Our results were similar for the absolute number of events as well as for adjusted HRs. We could not assess the impact of duration, dosage, and type of immunosuppressant drugs on outcomes of PD due to a lack of data.

Our review has some limitations. First, we obtained data from retrospective observational studies only, and their inherent bias may have influenced our results. Second, the total number of studies in our review was low, and not all studies reported every outcome of interest. Third, the outcomes in the studies may have been influenced by many known and unknown confounding variables. Baseline matchings for study variables were conducted only in a few studies. Most studies failed to report multivariable-adjusted HRs to independently assess the outcomes. This has important repercussions as outcomes data like technique failure and mortality can be influenced by several factors. There would have been bias in the choice of the initial dialysis technique depending upon many factors like functioning arterio-venous fistula, prior history of peritonitis, patient preference, etc. For evaluating mortality, the comparative group may not be ideal as there may be patients in the nTx group who may never undergo a transplant due to associated comorbidities. Lastly, data from the included studies were sourced over a long period. Changes in institutional practices concerning patient management, infection control protocols, PD techniques, and others were not considered and these may have skewed the study results. Furthermore, it is important to note that all outcomes in our analysis are time based events and the length of follow-up is an important factor while comparing outcomes data. The included studies varied significantly in the follow-up period and were unable to conduct a sub-group analysis for the same. This limitation should be considered while interpreting the results.

Nevertheless, our review is the first one to present pooled outcomes of patients with transplant failure initiating PD compared with those of transplant-naïve patients. We present a qualitative as well as a quantitative analysis for the readers. We pooled both absolute events and adjusted HRs of the outcomes to provide comprehensive evidence in our meta-analysis.

To conclude, the evidence from retrospective observational studies indicates that kidney transplant failure patients initiating PD do not have increased risks of mortality, technique failure, or peritonitis as compared to transplant-naïve patients initiating PD. Further studies should assess the impact of prior and ongoing immunosuppression therapy on PD outcomes of patients with kidney transplant failure. Future studies should also assess short-term and long-term outcomes separately to provide better evidence.

## Supplementary Material

Supplemental Material

Supplemental Material

Supplemental Material

Supplemental Material

Supplemental Material

Supplemental Material

Supplemental Material

Supplemental Material
