# Supplementary material for: Comparison of outcomes of peritoneal dialysis between patients after failed kidney transplant and transplant-naïve patients: a meta-analysis of observational studies
Source: Ren Fail. 2021 Apr 26;43(1):698–708. doi: 10.1080/0886022X.2021.1914659 (PMC8079072; doi:10.1080/0886022X.2021.1914659)
Supplement: Supplemental Material [file IRNF_A_1914659_SM0647.pdf]

Table S1: Search strategy and results of PubMed database

Search number	Query	Search Details	Results
**1**	((kidney transplant) AND (failure)) AND (peritoneal dialysis)	("kidney transplantation"[MeSH Terms] OR ("kidney"[All Fields] AND "transplantation"[All Fields]) OR "kidney transplantation"[All Fields] OR ("kidney"[All Fields] AND "transplant"[All Fields]) OR "kidney transplant"[All Fields]) AND ("failure"[All Fields] OR "failures"[All Fields]) AND ("peritoneal dialysis"[MeSH Terms] OR ("peritoneal"[All Fields] AND "dialysis"[All Fields]) OR "peritoneal dialysis"[All Fields])	3,386
**2**	(failed kidney transplant) AND (peritoneal dialysis)	("failed"[All Fields] OR "failing"[All Fields] OR "failings"[All Fields] OR "fails"[All Fields]) AND ("kidney transplantation"[MeSH Terms] OR ("kidney"[All Fields] AND "transplantation"[All Fields]) OR "kidney transplantation"[All Fields] OR ("kidney"[All Fields] AND "transplant"[All Fields]) OR "kidney transplant"[All Fields]) AND ("peritoneal dialysis"[MeSH Terms] OR ("peritoneal"[All Fields] AND "dialysis"[All Fields]) OR "peritoneal dialysis"[All Fields])	140
**3**	(prior kidney transplant) AND (peritoneal dialysis)	("prior"[All Fields] OR "priors"[All Fields]) AND ("kidney transplantation"[MeSH Terms] OR ("kidney"[All Fields] AND "transplantation"[All Fields]) OR "kidney transplantation"[All Fields] OR ("kidney"[All Fields] AND "transplant"[All Fields]) OR "kidney transplant"[All Fields]) AND ("peritoneal dialysis"[MeSH Terms] OR ("peritoneal"[All Fields] AND "dialysis"[All Fields]) OR "peritoneal dialysis"[All Fields])	252
**4**	(failed kidney transplant) AND (renal replacement therapy)	("failed"[All Fields] OR "failing"[All Fields] OR "failings"[All Fields] OR "fails"[All Fields]) AND ("kidney transplantation"[MeSH Terms] OR ("kidney"[All Fields] AND "transplantation"[All Fields]) OR "kidney transplantation"[All Fields] OR ("kidney"[All Fields] AND "transplant"[All Fields]) OR "kidney transplant"[All Fields]) AND ("renal replacement therapy"[MeSH Terms] OR ("renal"[All Fields] AND "replacement"[All Fields] AND "therapy"[All Fields]) OR "renal replacement therapy"[All Fields])	1,852
**5**	((transplant naive) AND (peritoneal dialysis)) AND (kidney transplant)	("transplantability"[All Fields] OR "transplantable"[All Fields] OR "transplantated"[All Fields] OR "transplantating"[All Fields] OR "transplantation"[MeSH Terms] OR "transplantation"[All Fields] OR "transplantations"[All Fields] OR "transplanted"[All Fields] OR "transplanting"[All Fields] OR "transplantation"[MeSH Subheading] OR "transplantation s"[All Fields] OR "transplanter"[All Fields] OR "transplanters"[All Fields] OR "transplantion"[All Fields] OR "transplants"[MeSH Terms] OR "transplants"[All Fields] OR "transplant"[All Fields]) AND ("naive"[All Fields] OR "naives"[All Fields]) AND ("peritoneal dialysis"[MeSH Terms] OR ("peritoneal"[All Fields] AND "dialysis"[All Fields]) OR "peritoneal dialysis"[All Fields]) AND ("kidney transplantation"[MeSH Terms] OR ("kidney"[All Fields] AND "transplantation"[All Fields]) OR "kidney transplantation"[All Fields] OR ("kidney"[All Fields] AND "transplant"[All Fields]) OR "kidney transplant"[All Fields])	12

6	(transplant naive) AND (peritoneal dialysis)	("transplantability"[All Fields] OR "transplantable"[All Fields] OR "transplantated"[All Fields] OR "transplantating"[All Fields] OR "transplantation"[MeSH Terms] OR "transplantation"[All Fields] OR "transplantations"[All Fields] OR "transplanted"[All Fields] OR "transplanting"[All Fields] OR "transplantation"[MeSH Subheading] OR "transplantation s"[All Fields] OR "transplanter"[All Fields] OR "transplanters"[All Fields] OR "transplantion"[All Fields] OR "transplants"[MeSH Terms] OR "transplants"[All Fields] OR "transplant"[All Fields]) AND ("naive"[All Fields] OR "naives"[All Fields]) AND ("peritoneal dialysis"[MeSH Terms] OR ("peritoneal"[All Fields] AND "dialysis"[All Fields]) OR "peritoneal dialysis"[All Fields])	14
7	(failed renal allograft) AND (peritoneal dialysis)	("failed"[All Fields] OR "failing"[All Fields] OR "failings"[All Fields] OR "fails"[All Fields]) AND ("renal"[All Fields] OR "renals"[All Fields]) AND ("allograft s"[All Fields] OR "allografted"[All Fields] OR "allografts"[MeSH Terms] OR "allografts"[All Fields] OR "allograft"[All Fields] OR "transplantation, homologous"[MeSH Terms] OR ("transplantation"[All Fields] AND "homologous"[All Fields]) OR "homologous transplantation"[All Fields] OR "allografting"[All Fields]) AND ("peritoneal dialysis"[MeSH Terms] OR ("peritoneal"[All Fields] AND "dialysis"[All Fields]) OR "peritoneal dialysis"[All Fields])	32
8	(prior renal allograft) AND (peritoneal dialysis)	("prior"[All Fields] OR "priors"[All Fields]) AND ("renal"[All Fields] OR "renals"[All Fields]) AND ("allograft s"[All Fields] OR "allografted"[All Fields] OR "allografts"[MeSH Terms] OR "allografts"[All Fields] OR "allograft"[All Fields] OR "transplantation, homologous"[MeSH Terms] OR ("transplantation"[All Fields] AND "homologous"[All Fields]) OR "homologous transplantation"[All Fields] OR "allografting"[All Fields]) AND ("peritoneal dialysis"[MeSH Terms] OR ("peritoneal"[All Fields] AND "dialysis"[All Fields]) OR "peritoneal dialysis"[All Fields])	32
