# Supplementary material for: Comparison of outcomes of peritoneal dialysis between patients after failed kidney transplant and transplant-naïve patients: a meta-analysis of observational studies
Source: Ren Fail. 2021 Apr 26;43(1):698–708. doi: 10.1080/0886022X.2021.1914659 (PMC8079072; doi:10.1080/0886022X.2021.1914659)
Supplement: Supplemental Material [file IRNF_A_1914659_SM0646.pdf]

Supplementary Table S2: Details of immunosuppressive drugs reported by included studies

Study	Data on immunosuppressant drugs in the study groups
De Costa 2020 [11]	87% patients in the Tx group received 5.0 (3.3–5.0) mg/day of prednisone for a period of 10.9 (2–21.2) months. Among these patients, 1 patient also received cyclosporine and mycophenolate sodium, which was withdrawn during the first month, and 1 patient was maintained on tacrolimus and azathioprine for 3 months. No patient in the nTx group received any immunosuppressive drug.
Chaudhri 2016 [14]	All patients in the Tx group were on prednisolone. Prednisolone dose at 90 days was 6.9mg/day and average dose remained essentially the same at 1 year (6.3mg/day). Only 2 (7%) patients were taking an anti-proliferative (azathioprine) on Day 90 after restarting PD (average dose of 75mg/day) and only 1 patient remained on this drug at 1 year. Calcineurin inhibitors were taken by 19 patients at Day 90 (14 were on ciclosporin, 5 on tacrolimus), but only 11 remained on this class of immunosuppressant at 1 year (7 on ciclosporin, 4 on tacrolimus).
Han 2015 [15]	All patients in the Tx group were on steroids, 4.9% on mycophenolate mofetil, 22% on calcineurin inhibitor. Daily dose of steroid was 2.2 (0.6-5.4) mg/day
Chen 2012 [17]	48.8% patients in the Tx group and 9.1% in the nTx group on immunosuppressant drugs
Duman 2004 [21]	All patients in the Tx group were on prednisolone for the first 90 days. 11.7% patients continued prednisolone during the study period. No patients on any other immunosuppressant drug. One patient in the nTx group was on prednisolone for rheumatoid arthritis.
Sasal 2001 [22]	92.8% patients in the Tx group remained on prednisone for a mean duration of 18.8 months. The mean dose of prednisone was 6.2 ± 2.1 mg daily. There were 3 nTx patients requiring immunosuppression for treatment of vasculitis/SLE, with a mean dose of 10 ± 4 mg/day for a mean of 24.7 months. Only 4 patients in the Tx group were treated with cyclosporine upon initiation of PD, which was discontinued in all cases within 4 months. Similarly, azathioprine was stopped in 2 patients within 3 months of starting PD.
Davies 2001 [23]	In the Tx group, steroids were withdrawn over a period of weeks to months, azathioprine was discontinued, and variable quantities of calcineurin inhibitors were prescribed.

Tx, failed transplant group; nTx, non-transplant group
