# Supplementary figures and images for: Comparison of outcomes of peritoneal dialysis between patients after failed kidney transplant and transplant-naïve patients: a meta-analysis of observational studies
Source: Ren Fail. 2021 Apr 26;43(1):698–708. doi: 10.1080/0886022X.2021.1914659 (PMC8079072; doi:10.1080/0886022X.2021.1914659)

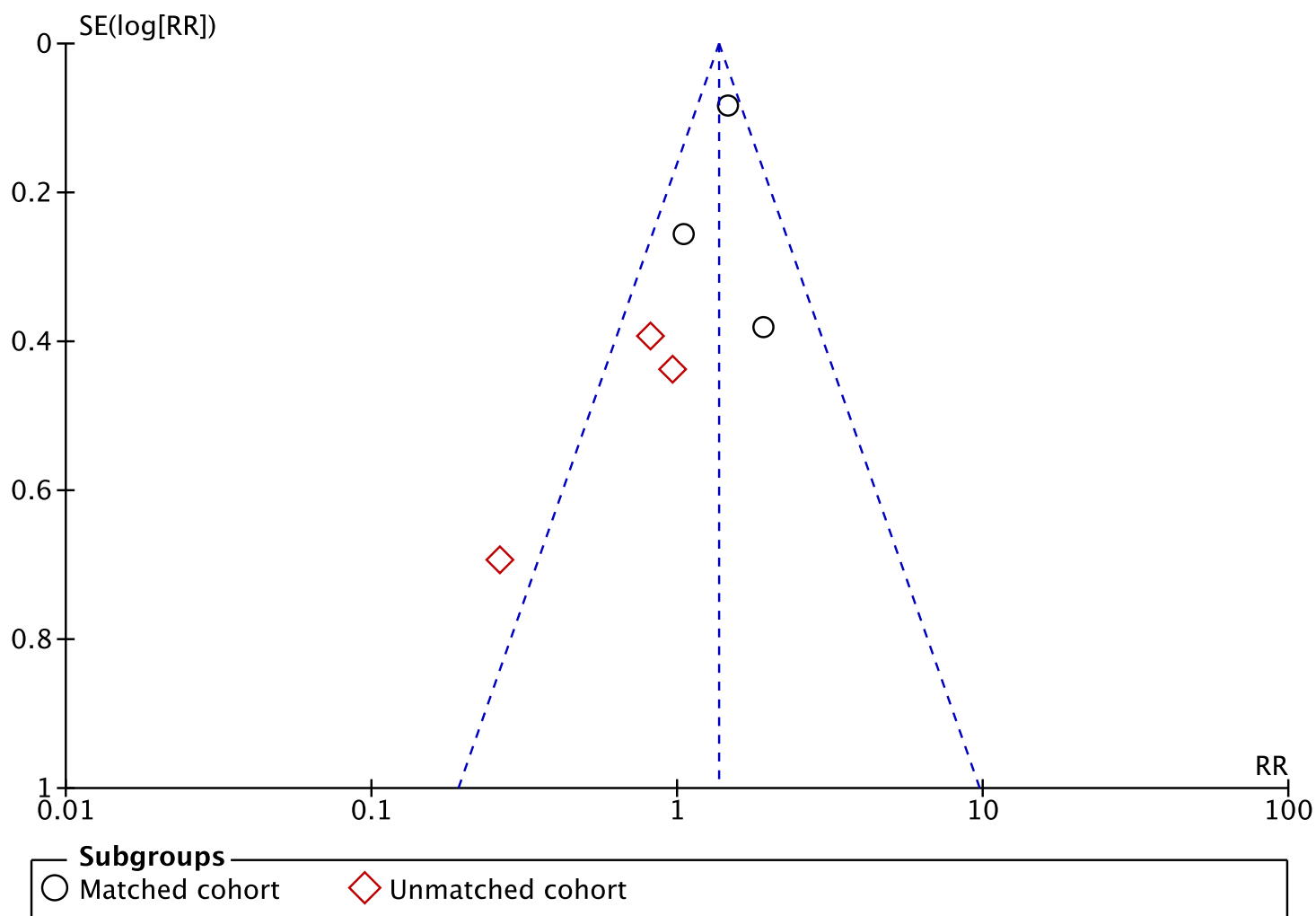

Supplement: Supplemental Material [file IRNF_A_1914659_SM0643.pdf]

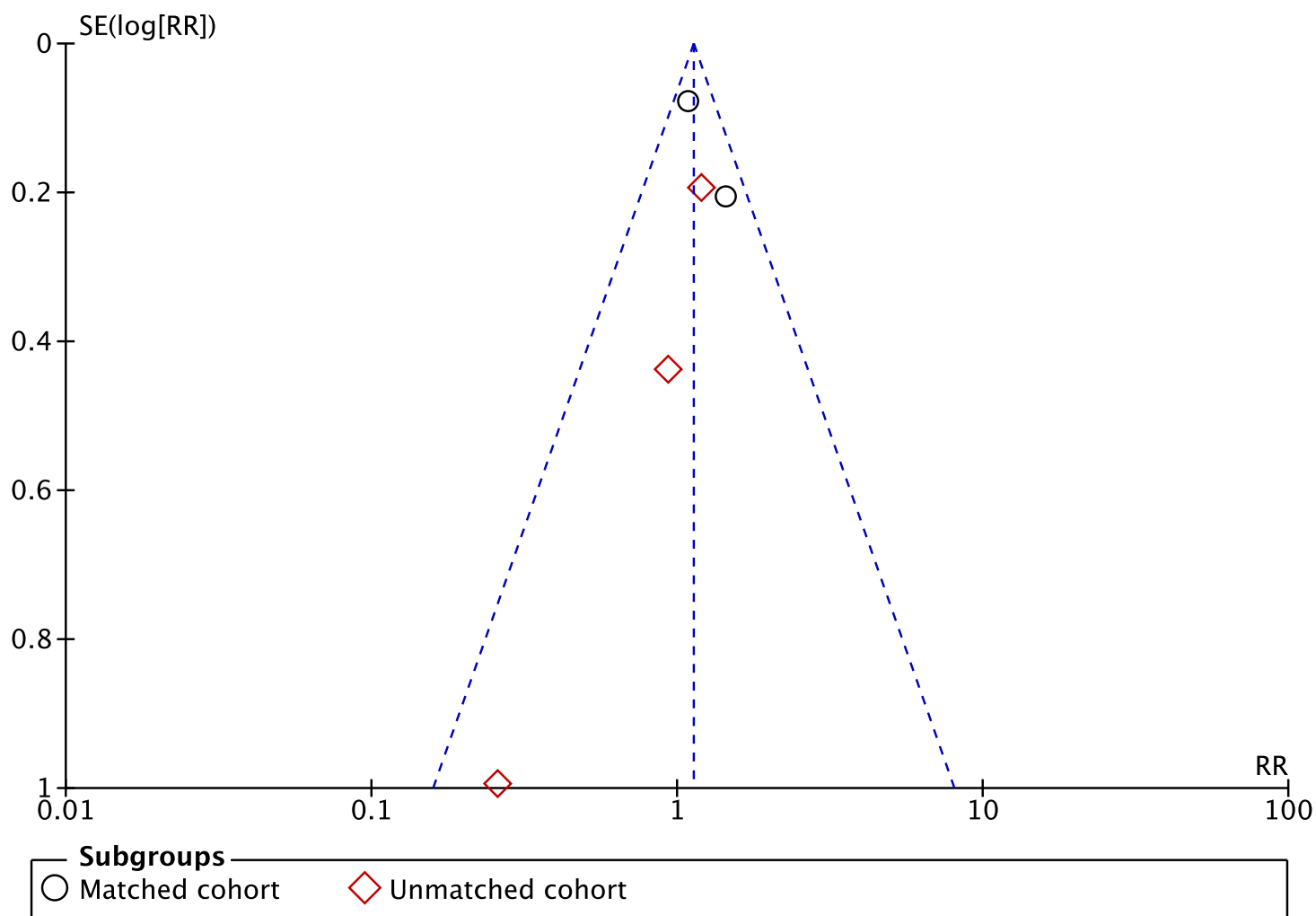

Supplement: Supplemental Material [file IRNF_A_1914659_SM0642.pdf]

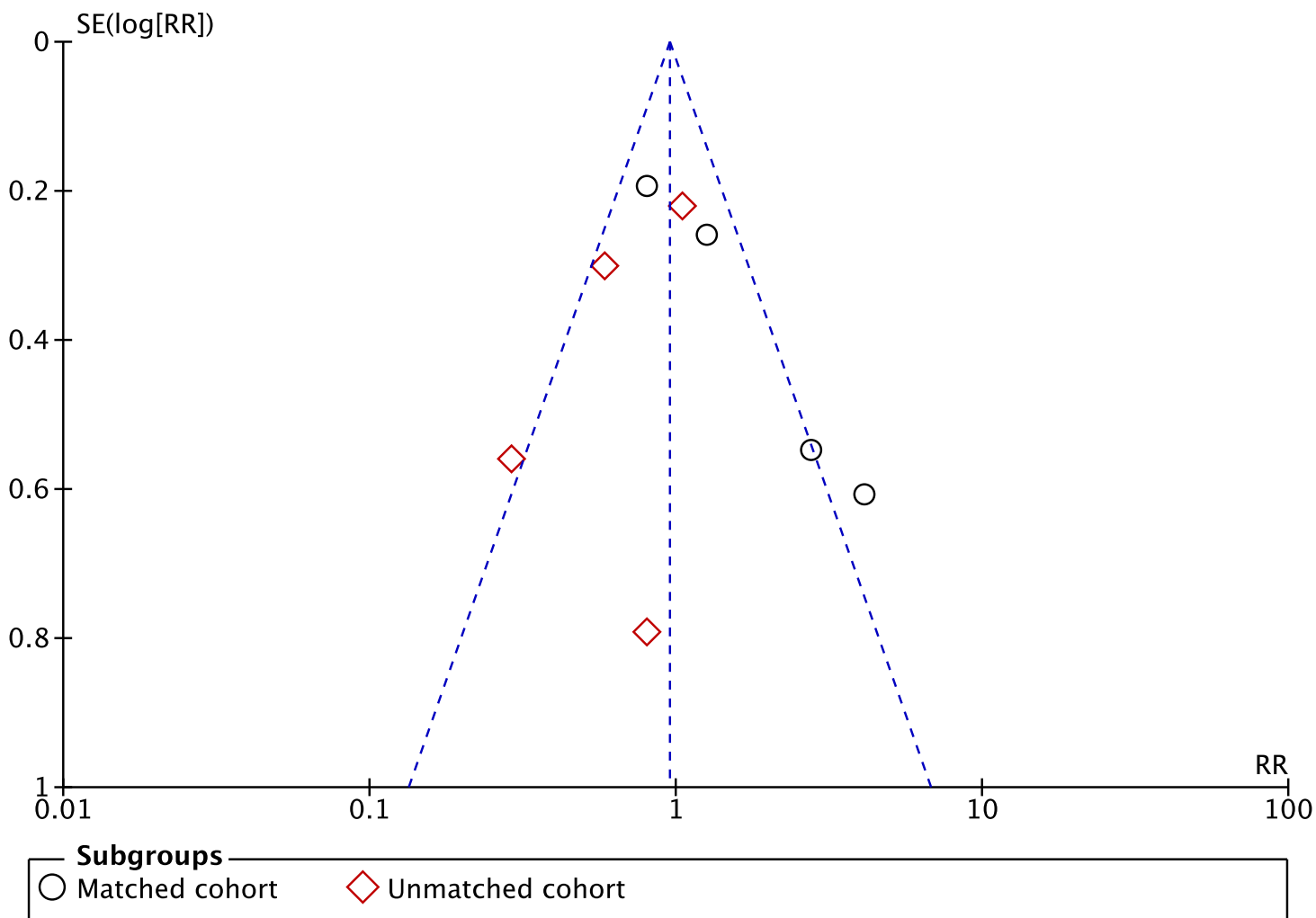

Supplement: Supplemental Material [file IRNF_A_1914659_SM0638.pdf]

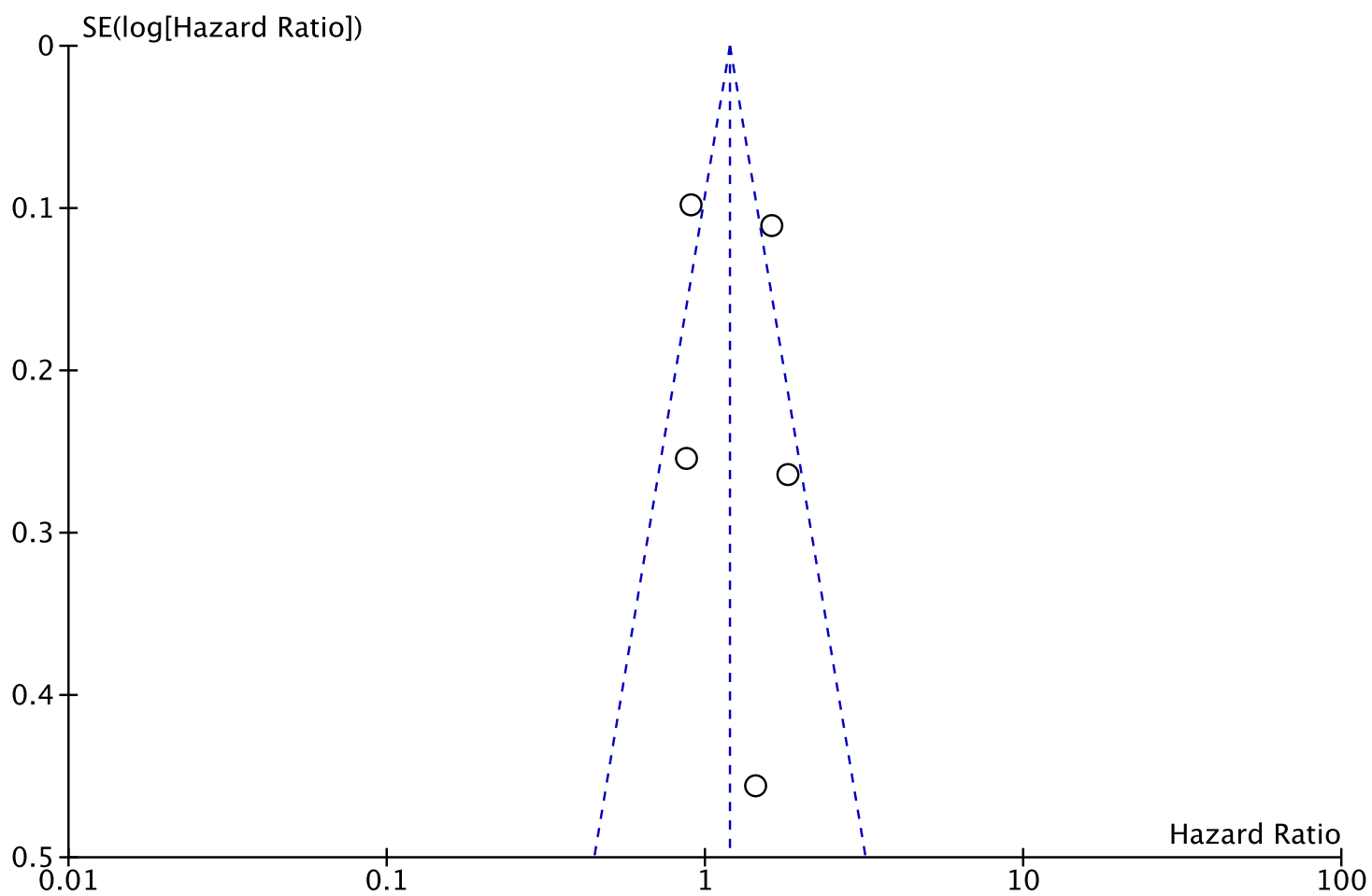

Supplement: Supplemental Material [file IRNF_A_1914659_SM0634.pdf]

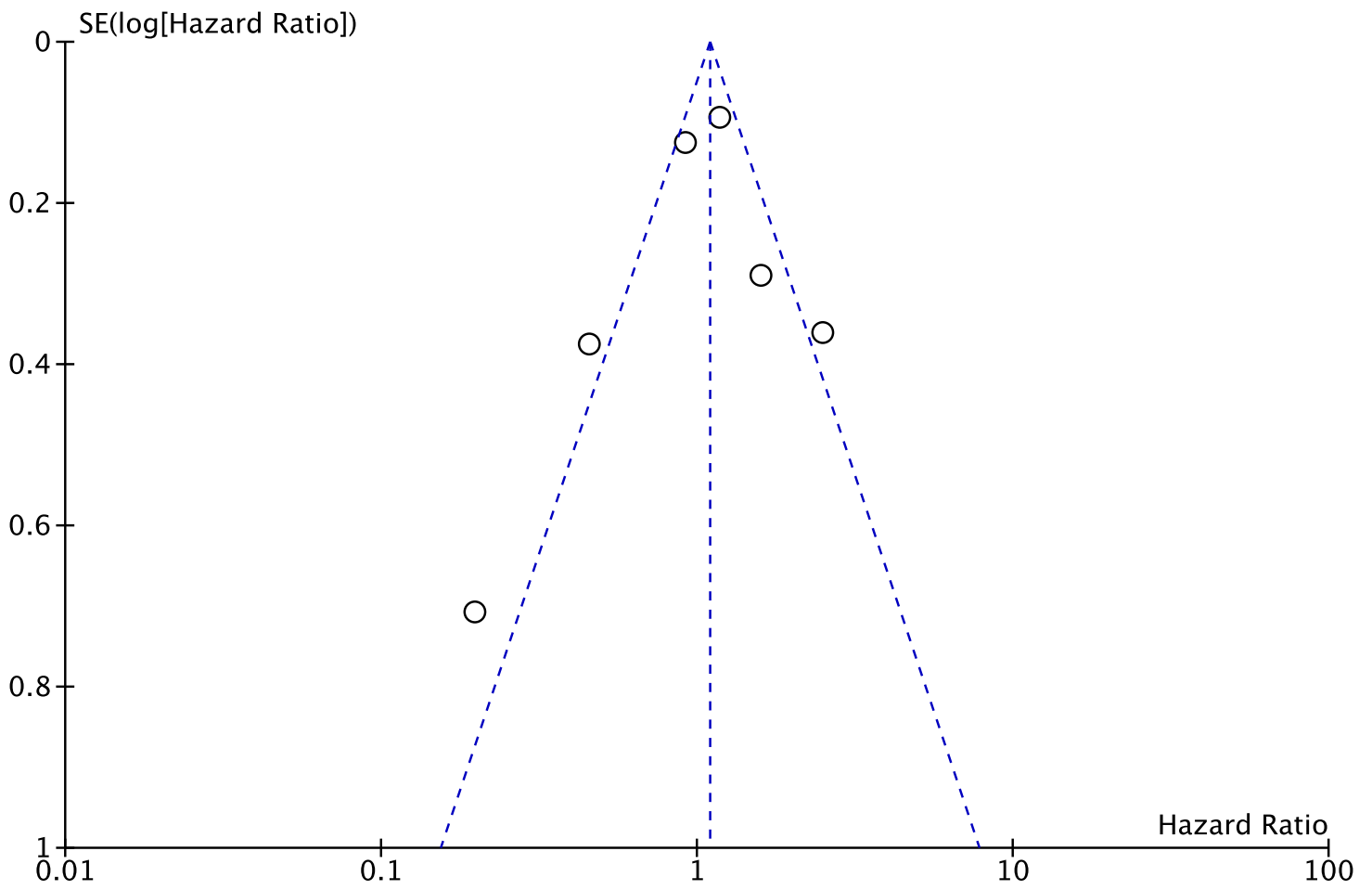

Supplement: Supplemental Material [file IRNF_A_1914659_SM0631.pdf]

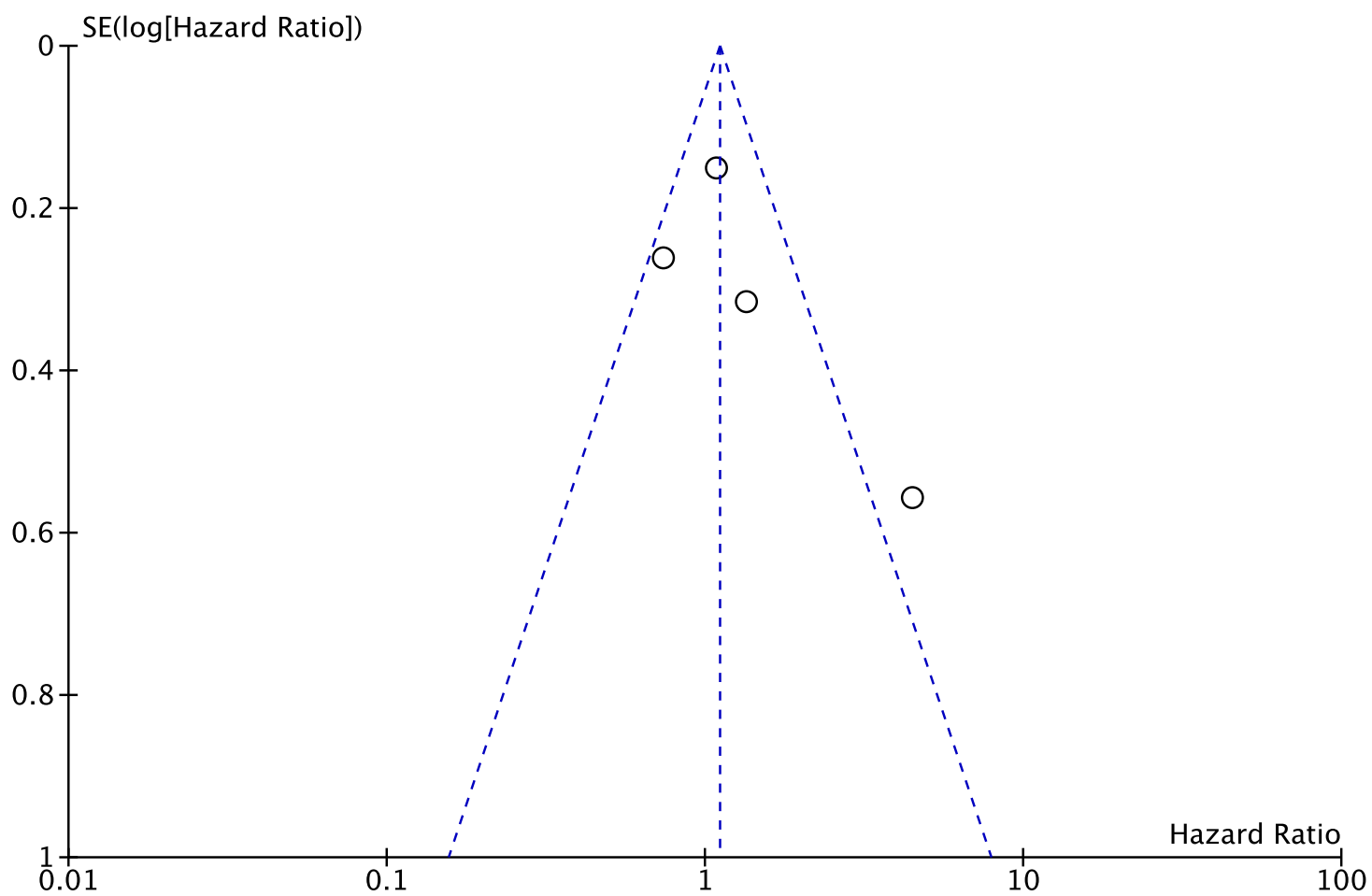

Supplement: Supplemental Material [file IRNF_A_1914659_SM0625.pdf]
